# Hepatic Transcriptome Analysis Provides New Insight into the Lipid-Reducing Effect of Dietary Taurine in High–Fat Fed Groupers (*Epinephelus coioides*)

**DOI:** 10.3390/metabo12070670

**Published:** 2022-07-20

**Authors:** Mingfan Chen, Fakai Bai, Tao Song, Xingjian Niu, Xuexi Wang, Kun Wang, Jidan Ye

**Affiliations:** 1 Xiamen Key Laboratory for Feed Quality Testing and Safety Evaluation, Fisheries College, Jimei University, Xiamen 361021, China; 202111908029@jmu.edu.cn (M.C.); 201911908010@jmu.edu.cn (F.B.); 202011908003@jmu.edu.cn (T.S.); niuqinjian@neau.edu.cn (X.N.); wangkun@jmu.edu.cn (K.W.); 2 Key Laboratory of Marine Biotechnology of Fujian Province, College of Marine Sciences, Fujian Agriculture and Forestry University, Fuzhou 350002, China or wangxuexi@fafu.edu.cn

**Keywords:** taurine, fat metabolism, liver fat accumulation, RNA–Seq, *Epinephelus coioides*

## Abstract

A transcriptome analysis was conducted to provide the first detailed overview of dietary taurine intervention on liver lipid accumulation caused by high–fat in groupers. After an eight-week feeding, the fish fed 15% fat diet (High–fat diet) had higher liver lipid contents vs. fish fed 10% fat diet (Control diet). 15% fat diet with 1% taurine (Taurine diet) improved weight gain and feed utilization, and decreased hepatosomatic index and liver lipid contents vs. the High–fat diet. In the comparison of the Control vs. High–fat groups, a total of 160 differentially expressed genes (DEGs) were identified, of which up- and down-regulated genes were 72 and 88, respectively. There were 49 identified DEGs with 26 and 23 of up- and down-regulated in the comparison to High–fat vs. Taurine. Several key genes, such as cysteine dioxygenase (*CDO1*), ADP–ribosylation factor 1/2 (*ARF1_2*), sodium/potassium–transporting ATPase subunit alpha (*ATP1A*), carnitine/acylcarnitine translocase (*CACT*), and calcium/calmodulin–dependent protein kinase II (*CAMK*) were obtained by enrichment for the above DEGs. These genes were enriched in taurine and hypotaurine metabolism, bile secretion, insulin secretion, phospholipase D signaling pathway, and thermogenesis pathways, respectively. The present study will also provide a new insight into the nutritional physiological function of taurine in farmed fish.

## 1. Introduction

Taurine is a sulfur−containing amino acid which is the most abundant free amino acid in animals [1]. It is clear that dietary taurine administration can reduce peripheral cholesterol and visceral lipid accumulation of rats and humans by enhancing rate–limiting enzyme activity of cholesterol 7α–hydroxylase in the liver, promote the synthesis of cholic acid from cholesterol, and increase the excretion of fecal cholesterol [2,3,4]. Taurine is a conditionally essential amino acid for most cultured fish [5]. It plays a range of key roles in fish physiology, including functions in bile acid conjugation, immune regulation, osmoregulation, antioxidation, nervous system development, and regeneration [6,7]. Moreover, the lipid-reducing effect of dietary taurine also occurred in different tissues of many farmed fish [8,9,10,11].

At present, one of the particularly concerning issues is fatty liver syndrome caused by widespread use of high–fat feed in intensive aquaculture for the purpose of protein-sparing and feed utilization [12,13]. Despite these benefits, excessive fat intake does cause growth retardation [14,15] and other undesirable effects, such as visceral lipid accumulation and fatty liver [16,17,18,19], accompanied by apoptosis and declined immune function [19,20,21]. Therefore, fatty liver induced by high–fat feeding has become a typical chronic liver disease which is closely associated with nutritional metabolic syndrome in intensive fish farming. There is an urgent need to find a suitable way to solve the problem of fatty livers, which represents a threat to aquaculture production. In the light of the lipid−reducing effect of taurine mentioned above, dietary taurine administration may therefore be a promising way to attenuate the adverse effects caused by high–fat diets.

The orange–spotted grouper (*Epinephelus coioides*) has become an economically important mariculture carnivorous fish species in Southeast Asian countries, including China [22,23]. Previous studies showed that dietary taurine supplementation can attenuate the tissue lipid accumulation of groupers [11,24]. However, the underlying mechanism involved in the regulation of lipid metabolism still remains unclear. Recent studies have shown that dietary taurine supplementation in 15% fat feed could reduce lipid accumulation through reducing the contents of triglyceride molecules containing 18:2n–6 at the *sn*–*2* and *sn*–*3* positions [25] and through accelerating lipid absorption of taurine−conjugated bile acids and fatty acid β-oxidation, and inhibiting lipogenesis [26]. However, determining how dietary taurine modulates lipid metabolism of fish at the transcriptional level has not yet been investigated. In the present study, three experimental diets (10% fat, 15% fat and 15% fat with 1% taurine) were formulated to investigate the lipid–reducing effect of dietary taurine supplementation in regard to taurine-mediated changes of key genes.

## 2. Results

### 2.1. Growth Performance and Tissue Lipid Contents

The growth performance of groupers is shown in Table 1. When the dietary lipid level increased from 10% to 15%, weight gain, feed conversion ratio and hepatosomatic index of the Control and High–fat groups were not different (*p* > 0.05). However, when 15% lipid diet was added with 1% taurine, weight gain was significantly enhanced, whereas feed conversion ratio and hepatosomatic index were markedly reduced compared with High–fat group (*p* < 0.05).

The lipid contents in the liver and muscle samples in High–fat group were significantly higher (*p* < 0.05) than that in Control group. Fish of Taurine group had lower (*p* < 0.05) liver lipid contents vs those of High–fat group. However, there was no difference in muscle lipid content between High–fat and Taurine groups (*p* > 0.05).

### 2.2. Illumina Sequencing and De Novo Assembly

According to the sequencing results of the transcriptome assay, we obtained 399.25 million clean reads (Table S1), with Q20 at between 97.56% and 98.46%, Q30 at between 93.16% and 95.45%, and the GC content between 42.16% and 47.00%. From the assembly results, 99,634 unigenes were obtained with the N50 and N90 of unigenes being 653 and 243 bp respectively (Table 2). The length distribution of transcript and unigenes is shown in Figure S1. After short and low–quality sequences were excluded, 349,211 unigenes were identified and annotated by matching them against the five public databases (NR, SwissProt, PFAM, GO and KO), and 72,679 unigenes had at least one Blast hit against the public databases, yielding 72,125 annotated unigenes for NR (20.65%), 13,104 for SwissProt (3.75%), 22,814 for PFAM (6.53%), 6988 for GO (2.00%) and 5124 for KO (1.47%) (Table 3).

### 2.3. Identification of DEGs

**Figure 1:**
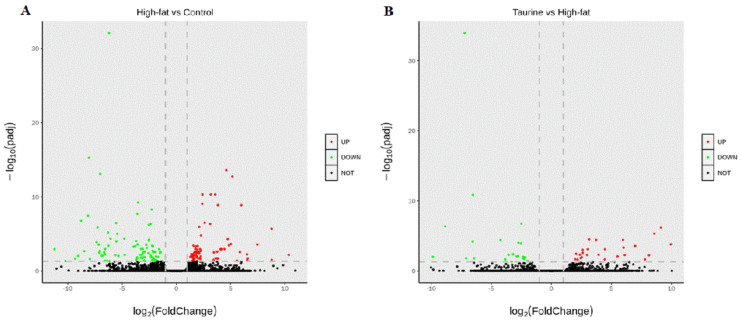
Comparison of DEGs among liver transcriptome of *E. coioides* of the Control and the High-fat groups, and of the High–fat and the Taurine groups. Control, control diet; High–fat, 15% fat diet; Taurine, 15% fat diet with 1% taurine. (**A**)—volcano plot of DEGs of High-fat vs. Control group; (**B**)—volcano plot of DEGs of Taurine vs. High–fat group.

To identify DEGs in liver samples of grouper after feeding trial, three digital gene expression libraries from the Control, High–fat, and Taurine groups were constructed (Figure 1). There were 160 DEGs identified in the comparison between the High–fat and Control groups, of which 72 were upregulated and 88 were downregulated. In the comparison between the Taurine and High–fat groups, 49 DEGs were identified, of which 26 were upregulated and the rest were downregulated.

### 2.4. Function Annotation and Analysis for Unigenes

**Figure 2:**
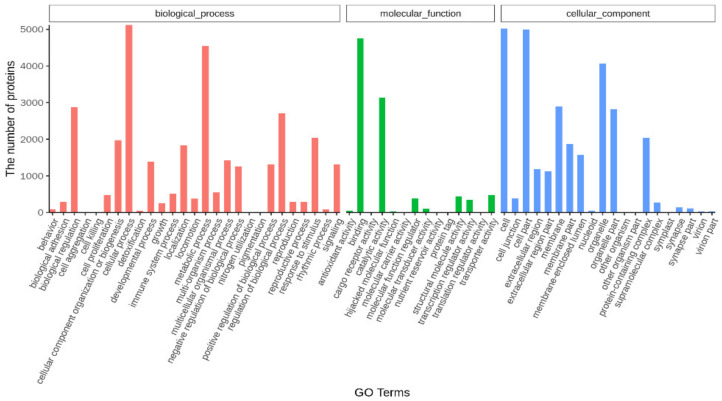
Classification of the unigenes and annotation of differentially expressed genes (DEGs) of liver samples of *E. coioides* based on enrichment analysis of gene ontology. All DEGs were enriched into three categorizations: biological processes, cellular components and molecular function categories.

Based upon gene ontology (GO) classification, 6988 (2.00%) unigenes were mapped and clustered into biological processes, cellular components and molecular function categories. In the classification of biological processes, the cellular process and metabolic process occur most frequently. In the cellular components, most unigenes were classified into a cell and cell part. With regard to molecular function, most unigenes were clustered into binding and catalytic activity categories. The GO annotation statistics of Unigenes is shown in Figure 2.

**Figure 3:**
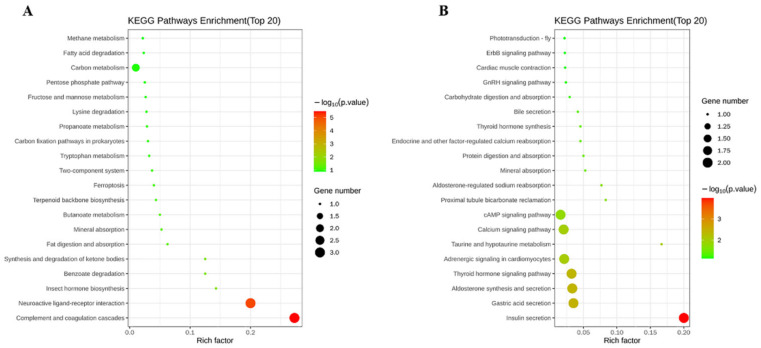
Bubble chart of significantly enriched KEGG pathways in the differentially expressed genes (DEGs) of liver samples of *E. coioides* between the Control and High–fat groups, and between High–fat and Taurine groups. (**A**)—pathway enrichment of DEGs of High–fat vs. Control groups; (**B**)—pathway enrichment of DEGs of High–fat vs. Taurine groups. The vertical axis represents the pathway categories, the horizontal axis shows the enrichment factor. The point size shows the number of DEGs enriched in the KEGG pathway. The point color shows different Q values as indicated on the right.

Upon KEGG enrichment analysis, 160 differentially expressed genes (DEGs) in the liver samples were identified in the comparison with the High–fat vs. the Control groups, of which 72 genes were upregulated and 88 genes were downregulated. The typical pathways of DEGs for enrichment were complement and coagulation cascades, respectively (Figure 3). The comparison of Taurine and High–fat groups had 49 DEGs (26 genes were upregulated and the rest were downregulated) for enrichment in the liver. The insulin secretion was the most enriched pathway of DEGs (Figure 3).

### 2.5. Signaling Pathway Network Related to Lipid Metabolism

**Figure 4:**
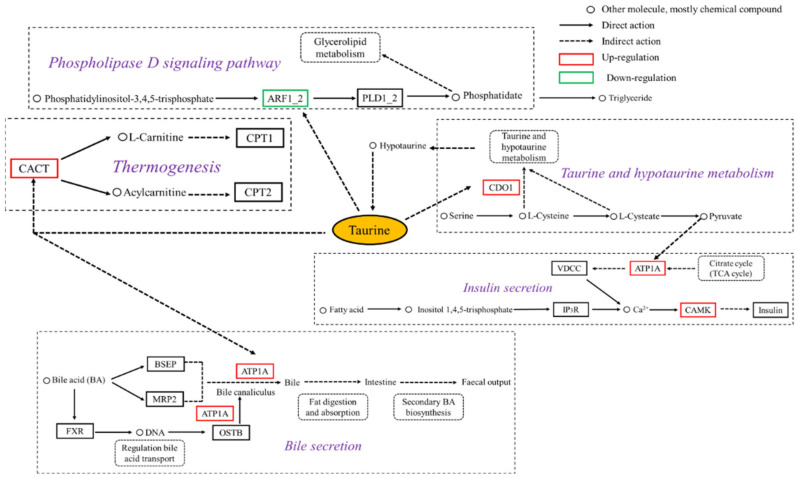
Signal pathway network diagram of taurine–mediated lipid metabolism in the liver of *E. coioides*. Abbreviations: AACT, carnitine/acylcarnitine translocase; ARF1_2, ADP–ribosylation factor 1/2; ATP1*α*, sodium/potassium–transporting ATPase subunit alpha; CAMK, calmodulin–dependent protein kinase II; CDO1, cysteine dioxygenase; GAPN, glyceraldehyde-3-phosphate dehydrogenase; GK, glucokinase; PDK1, pyruvate dehydrogenase kinase isozyme 2; PLCD, phosphatidylinositol phospholipase C.

According to the above analysis results, the DEGs were annotated as key genes such as cysteine dioxygenase (*CDO1*), ADP–ribosylation factor 1/2 (*ARF1_2*), sodium/potassium–transporting ATPase subunit alpha (ATP1α) in the carnitine/acylcarnitine translocase (CACT) and calcium/calmodulin-dependent protein kinase II (CAMK). These key genes were high–fat induced lipid metabolism genes regulated by dietary taurine in groupers. Several signaling pathways of taurine–mediated lipid metabolism were clustered in Table S2 and mainly included taurine and hypotaurine metabolism, primary bile secretion, insulin secretion, phospholipase D signaling pathway, phosphatidylinositol signaling system, inositol phosphate metabolism and calcium signaling pathway. Furthermore, the signaling pathway network related to taurine–mediated lipid metabolism in the liver was constructed by KEGG functional enrichment pathway conjoint analysis and is presented in Figure 4.

### 2.6. qRT–PCR Validation of DEGs

**Figure 5:**
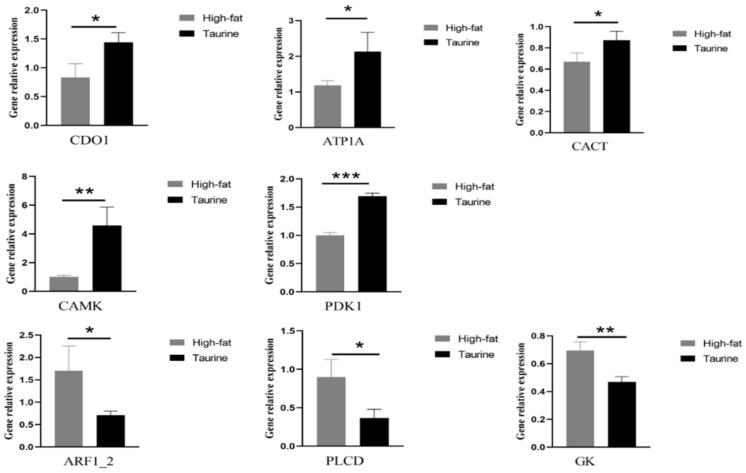
Validation of RNA–seq data using qRT–PCR in the liver of *E. coioides* in High–fat and Taurine groups. To validate the data from RNA–seq analysis, relative mRNA levels of eight selected differentially expressed genes (*ARF1_2*, *GK*, *ATP1α*, *CAMK*, *CDO1*, *CACT*, *PDK1*, and *PLCD*) from the liver samples of Control, High–fat and Taurine groups were examined by RT–qPCR. mRNA levels are presented as –fold changes when compared with the Control group after normalization against β-actin. The relative mRNA levels from the RNA–seq analysis were calculated as FPKM values. High–fat, 15% fat diet; Taurine, 15% fat diet with 1% taurine. Student’s *t*–test was applied for comparison of High–fat vs. Taurine diets. Values are means ± SEM (*n* = 3). Asterisks (*, ** and ***) represent significant differences with *p* < 0.05, *p* < 0.01 and *p* < 0.001 respectively.

To validate the reliability of RNA–Seq data, eight randomly selected DEG expression profiles in taurine and high fat comparison groups samples were examined by qRT–PCR. As shown in Figure 5, the fold–changes obtained by qRT–PCR were consistent with the values obtained by RNA–seq for the six selected genes (*ARF1_2*, *GK*, *ATP1α*, *CAMK*, *CDO1*, and *CACT*), suggesting our RNA-Seq data and the results based on RNA–Seq data analysis were reliable.

## 3. Discussion

The results of the present study showed that the growth and feed utilization of groupers did not differ when the dietary fat was increased from 10% to 15%, which indicates that the fish species has a certain degree of tolerance to a higher fat diet. Similar results were observed in previous studies of black sea bream (*Acanthopagrus schlegelii*) [27] and large yellow croaker (*Larimichthys crocea*) [28]. However, high–fat diets increased liver lipid contents vs. control diets in this study and previous studies on many farmed fishes [16,17,18,20,28,29,30], indicating that feeding high–fat diet led to an increase in liver lipid accumulation of fish.

Interestingly, taurine addition in high-fat diets not only promoted both fish growth and feed utilization in the study and previous studies on many farmed fishes [31,32,33,34,35], but also reduced liver lipid content and/or hepatosomatic index of farmed fishes [10,11,36,37,38,39,40]. All of these results indicate a significant effect of taurine on reducing liver lipid. In view of the lipid–reducing effect of taurine on fish above mentioned, the next objective of this study was to investigate how high–fat diet does affect liver lipid deposition and to explore the lipid-reducing effect of dietary taurine intervention in relation to taurine–mediated changes of gene expression at the transcriptome level.

Herein, we present the first transcriptomic analysis of taurine intervention on the liver lipid metabolism of high–fat fed groupers in an attempt to understand the regulatory mechanism of taurine on liver lipid deposition caused by high–fat feeding. The DEGs were then screened out after the unigenes were mapped to five public databases (NR, SwissProt, PFAM, GO and KO) by the NCBI, and had at least one Blast hit against the public databases. A total of 160 DEGs were identified in the comparison of High–fat and Control groups. In the comparison of High–fat and Taurine groups, a total of 49 DEGs were identified. GO annotation and KEGG pathway analysis of DEGs in the comparison of Taurine and High–fat groups showed that the DEGs were enriched in primary bile secretion, insulin secretion, phospholipase D signaling pathway and thermogenesis pathways. The discovery of these genes and signaling pathways should contribute to a better understanding of the molecular mechanism of regulation of taurine in fish lipid metabolism.

The cysteine sulfite dependent pathway is the main route of taurine biosynthesis in mammals, in which CDO1, as the rate–limiting enzyme, can produce hypotaurine through oxidation and decarboxylation, then hypotaurine is oxidized to form taurine [41]. Fish also have the capability for biosynthesis of taurine by CDO1 [6]. The activity of CDO1 in fish liver is generally higher than that in other tissues [5]. Dietary taurine addition was reported to upregulate the gene expression of *CDO1* in tissues of Atlantic bluefin tuna (*Thunnus thynnus*) [42], but did not affect its expression in the liver of tiger puffer (*Takifugu rubripes*) [43] and European bass (*Dicentrarchus labrax*) [8]. The dietary taurine promotes liver *CDO1* expression level in a dose–dependent manner [37]. The above results show that the ability of fish to synthesize taurine varies between fish species [37]. In this study, there was no difference in expression level of *CDO1* between Control and High–fat diets, but the Taurine diet resulted in an upregulation in gene expression of *CDO1* compared with the High–fat diet. The *CDO1* gene was enriched in the taurine and hypotaurine metabolic pathway. One possible reason for this is the fact that feeding high–fat diet may increase the demand for taurine of groupers. This indicates that taurine exerts the effect of reducing liver fat by affecting the synthesis and metabolism of taurine.

In the present study, dietary taurine supplementation downregulated the expression of ADP–ribosylation factor (ARF) gene *ARF1_2* compared with the High–fat group. The *ARF1_2* gene was enriched into phospholipase D signaling pathway. The *ARF1_2* gene is a member of the Ras superfamily, and participates in vesicular trafficking and regulating the activation of phospholipase D (PLD), which plays an important role in intracellular signal transduction and substance transport [44]. PLD catalyzes not only the hydrolysis of the phosphodiester bond of glycerophospholipids to generate phosphatidic acid (PA), but also a transphosphatidylation reaction to produce phosphatidylethanol [45]. The PLD activated by ARF1_2 hydrolyzed phosphatidylcholine to produce more precursor PA required for triglyceride synthesis [46,47]. Therefore, dietary taurine supplementation may reduce the synthesis of triglycerides in the liver of fish through downregulating *ARF1_2* gene expression.

As a signal molecule, bile acids are able to regulate their own enterohepatic circulation by affecting transcription of the genes critically involved in transport and metabolism [48]. The farnesoid X receptor (FXR) is activated by bile acids [49]. The activated FXR triggers the transcriptional synthesis of bile salt export pump (BSEP) [50]. Due to the high affinity of BSEP to bile acids, the bile acids in the form of complex bile acids and BSEP are transported from hepatocytes to the intestine with the help of the energy generated by ATP hydrolysis. BSEP is therefore considered to be the most important ATP dependent bile acid transporter in the liver [51]. The bile acid metabolism and transport are achieved through the chain reaction triggered by FXR and its downstream BSEP [50,52], so as to maintain the glycolipid metabolic homeostasis of fish [18,53]. Dietary taurine administration ameliorates Na^+^/K^+^ATPase impairment in the retina of diabetic rats [54] and promotes gill Na^+^/K^+^ATPase activity of rainbow trout (*Oncorhynchus mykiss*) [55]. In this study, after dietary taurine intervention in the High–fat group, *ATP1α* gene expression was upregulated and enriched into the bile secretion pathway. Therefore, dietary taurine administration accelerates bile acid transport and metabolism through upregulating *ATP1α* gene expression.

Lipid metabolic disorder is associated with abnormal energy metabolism, including gluconeogenesis, glycogenolysis, and the TCA cycle. GK is a rate–limiting enzyme required for glucose metabolism in the liver and the regulation of insulin secretion from islets [56]. Hepatic GK enzyme activity of glycolysis pathway is stimulated by glucose uptake [57], contributing an increase in liver glycogen content to maintain glucose homeostasis. In contrast, the downregulation of hepatic *GK* gene and protein expression was accompanied by reduced hepatic glycogen synthesis in type 2 diabetic rats [58]. The findings of the present study showed that dietary taurine supplementation could inhibit the glycolysis pathway as well as the biosynthesis of pyruvate, which is the final metabolites of glycolysis and the substrate for lipid synthesis de novo. Therefore, the lipid–reducing effect of taurine on fish can also be indirectly achieved by inhibiting the biosynthesis of substrate of lipogenesis de novo via down–regulating the gene expression in the glycolysis pathway.

The activation of CAMK II was involved in the hypertonicity–induced upregulation of human taurine transporter [59], enhancing insulin secretion [60]. In this study, the expression of gene *CAMK* (Ca^2+^/Calmodulin kinase II) was up-regulated after dietary taurine intervention, and enriched into the insulin secretion signal pathway. Taurine can promote insulin secretion through the interaction between taurine and ATP sensitive K^+^ (KATP) channels [61,62].

Insulin is an important endocrine hormone. It promotes free fatty acid and cholesterol uptake, reduces lipolysis, and increases lipogenesis in fish [63] through regulating several enzymes involved in lipogenesis and lipolysis, as well as transcription factors regulating the expression of such enzymes [64,65]. High–fat diets caused lipid metabolism disorder, insulin resistance, and liver steatosis in mammals [66], and liver steatosis was positively associated with insulin resistance in nonalcoholic fatty liver disease [67]. Dietary taurine administration can increase the insulin sensitivity of mammals [68,69]. Therefore, dietary taurine may promote insulin secretion through up regulating the expression of *CAMK* gene. On the one hand, it may inhibit insulin resistance caused by a high–fat diet. On the other hand, it is conducive to the decomposition of peripheral free fatty acids and cholesterol into the liver, thus reducing peripheral fat deposition. However, the relevant results here still need further study in this regard.

Liver lipid homeostasis regulation involves a complex interaction of triglycerides present in hepatocytes including fatty acid uptake, de novo lipogenesis, fatty acid β-oxidation, and fatty acid export [66,70,71]. Abnormal liver fat accumulation is often accompanied by excessive production of reactive oxygen free radicals and intermediates with lipotoxicity such as diacylglycerol as well as endoplasmic reticulum stress, resulting in lipid metabolism disorder [71,72]. Mitochondria are recognized as the main organelles of fatty acid β-oxidation. The fatty acid β-oxidation is regulated by gene *CACT* [73]. The transcriptional level of *CACT* gene was up–regulated by fish oil both in rats and fish compared with beef tallow or lard oil [74,75]. In this study, dietary taurine intervention resulted in an upregulation of the *CACT* gene expression. The *CACT* gene was enriched into the thermogenesis pathway. This indicates that up–regulated *CACT* gene expression caused by dietary taurine intervention promotes fatty acid transport to mitochondria and fatty acid β-oxidation, thereby reducing lipid deposition in the liver of groupers.

## 4. Materials and Methods

### 4.1. Experimental Diets

The optimal dietary levels of lipid and taurine were at about 10% and 1%, respectively for the growth of *E. coioides* [76,77]. In this experiment, therefore, three isonitrogenous (46% crude protein) experimental diets were prepared using casein and gelatin without taurine (food grade) and shrimp meal as protein source, fish oil, soy oil and soy lecithin as lipid source, namely 10% fat det (control diet), 15% fat diet (high fat diet) and 15% fat + 1% taurine diet (Taurine diet). The ingredients and proximate composition of the three experimental diets are shown in Table 4. The experimental diets were produced according to the method described in detail previously [24].

### 4.2. Growth Trial

Juvenile groupers purchased from a local fish farm (Zhangpu county, Fujian province, China) were transported to the Aquaculture Experimental Center of Jimei University. They were fed a commercial feed for two weeks of acclimatization in two tanks (1000 L/tank) before the start of the experiment. A total of 270 fish with a similar size (an initial wet body weight of 10.5 ± 0.1 g) were randomly distributed into 9 fiberglass tanks at a stocking density of 30 fish per tank (300 L/tank), within a recirculating water aquaculture system connected with a circulation pump, biological filters, and an automatic temperature control device. The fish in triplicate tanks of each group were fed one of the three experimental diets to apparent satiation twice daily (8:30 and 18:30), across a feeding period of 8 weeks. Excess feed was collected 30 min after each meal, dried in a ventilated oven at 65 °C, and weighed for the calculation of feed intake. During the feeding period, water temperature was maintained at about 28.0 °C, dissolved oxygen level ranged between 6.1 ± 0.2 mg/L, and the ammonia nitrogen level was less than 0.2 mg/L.

### 4.3. Sample Collection

At the end of the 8-week feeding trial, the fish in each tank were caught and anesthetized with a dose of 100 mg/L solution of MS-222 (tricaine methane sulphonate, Sigma-Aldrich Shanghai Trading Co. Ltd., Shanghai, China), followed by fish count and batch–weighing, and recorded on a wet weight basis to determine percent weight gain and feed conversion ratio. Five fish were randomly caught from each tank and individually weighed. The liver was then removed after abdominal dissection, and weighed for the determination of hepatosomatic index. The liver samples were pooled by tank, frozen immediately in liquid nitrogen and stored at −20 °C for the analysis of fat content. For gene expression analysis, another three fish in each tank were randomly sampled and anesthetized. After drawing blood, liver samples were aseptically removed and quickly frozen with liquid nitrogen and stored at −80 °C.

### 4.4. Composition Analysis

Proximate composition of ingredients, diets, and tissue samples were determined according to standard methods [78]. Dry matter was determined by drying the samples in an oven at 105 °C to a constant weight. Crude protein was determined by the Kjeldahl method (N × 6.25) using Kjeltec TM 8400 Auto Sample Systems (Foss Teacher AB). The crude fat content was determined by the Soxtec extraction method by using Soxtec Avanti 2050 (Foss Teacher AB). Ash was measured in the residues of samples burned in a muffle furnace at 550 °C for 8 h.

Total fat of muscle and liver samples were extracted by homogenization in chloroform/methanol (2:1, *v*/*v*) solution and determined gravimetrically after drying a 5 mL aliquot under nitrogen [79]. For taurine determination [80], feed samples were hydrolysed in nitrogen–flushed glass vials using 6 mol/L HCl at 116 °C for 22 h, followed by centrifugation (1500 g, 4 °C, 15 min), and the supernatant was collected and analyzed using an automatic AA analyzer (Hitachi L8900, Tokyo, Japan).

### 4.5. RNA Extraction and cDNA Library Construction

Total RNA was extracted from the liver sample using TRIzol^®^ reagent (Magen) according to the manufacturer’s instructions. The A260/A280 absorbance ratios of RNA in the liver samples were detected to test the RNA purity by using NanoDrop ND–2000 spectrophotometer (Thermo Scientific, Waltham, MA, USA). The RIN value of sample RNA was detected to test the RNA quality of the samples by using Agilent Bioanalyzer 4150 (Agilent Technologies, Santa Clara, CA, USA). The cDNA library was constructed using the Illumina HiseqTM 2000 system (Illumina, San Diego, CA, USA) by APTBIO Co., Ltd. (Shanghai, China). Raw reads were filtered to remove low-quality sequences using the program written by APTBIO Co., Ltd.

### 4.6. Sequence Data Processing and Analysis

RNA–Seq de novo assembly was carried out by using Trinity software (http://trinityrnaseq.github.io/) (accessed on 27 July 2020). The longest transcripts were regarded as unigenes after removing repetitive assemblies. BLAST software (http://blast.ncbi.nlm.nih.gov/Blast.cgi/) (accessed on 27 July 2020) was used to align unigene sequences with the Non-Redundant Protein Sequence Database (NR, https://ftp.ncbi.nlm.nih.gov/blast/db/FASTA/) (accessed on 27 July 2020), Protein Families Database (Pfam, http://pfam.xfam.org/) (accessed on 27 July 2020), SwissProt protein Database (SwissProt, https://www.expasy.org/) (accessed on 27 July 2020), Kyoto Encyclopedia of Gene and Genomes Database (KEGG, http://www.kegg.jp/) (accessed on 27 July 2020), and Gene Ontology Database (GO, http://geneontology.org) (accessed on 27 July 2020). RESM software (http://deweylab.github.io/RSEM/) (accessed on 27 July 2020) was used to accurately map the sequencing reads to reference genomes. The expression level of each gene was calculated from the fragment per kilobase of exon model per million mapped read (FPKM) values [81].

### 4.7. Identification and Enrichment Analysis of Differentially Expressed Genes

DESeq2 software (https://bioconductor.org/packages/release/bioc/html/DESeq2.html) (accessed on 27 July 2020) was used to determine differentially expressed genes (DEGs), and unigenes with *p*-value < 0.05 and |log2 foldchange| > 1 were defined as DEGs. In the process of DEGs analysis, the recognized and effective Benjamin Hochberg method was used to correct the significance *p*-value obtained from the original hypothesis test. The corrected *p*-value, FDR (false discovery rate), is then used as the key index of DEG screening to reduce the false positive caused by independent statistical hypothesis test on the expression value of a large number of genes. The number of up- and down-regulated DEGs in the liver under different dietary treatments was obtained. Using Blast2go software (https://www.blast2go.com/) (accessed on 27 July 2020), the gene ontology (GO) annotation information of all DEGs was obtained, the GO function of DEGs was classified, and the molecular function, cell composition and biological process of target genes were described [82]. The pathway enrichment analysis was performed using online service tool KAAS (KEGG automatic annotation server). Fisher’s precision probability test was used to calculate the significance of enrichment of each gene in the pathway, so as to determine the corresponding significant signal transduction and metabolic pathways. The enrichment results of DEGs are displayed by KEGG enrichment scatter diagram.

### 4.8. Quantitative Real–Time PCR Analysis

The relative expression levels of eight selected DEGs were verified by quantitative reverse transcription PCR (qRT–PCR), so as to validate the gene expression data obtained by RNA–Seq. The eight pairs of primers were designed with Primer v. 5.0, with the β-actin gene of the fish used as the internal reference for the qPCR analysis (Table S3) and sent to jinweizhi Biotechnology Co., Ltd. (Suzhou, China) for synthesis. The qRT–PCR reactions were performed using ChamQ Universal SYBR qPCR Master Mix (Vazyme, Nanjing, China) on a Real–Time PCR Detection System (ABI 7500, Applied Biosystems, Waltham, MA, USA). The PCR cycling conditions were as follows: 95 °C for 30 s, followed by 40 cycles of 95 °C for 10 s and 60 °C for 30 s, and then cycles at 95 °C for 15 s, 60 °C for 60 s and 95 °C for 15 s. To check reproducibility, the qRT–PCR reaction for each sample was performed in four biological replicates. The relative expression of genes was calculated using the 2^−ΔΔCt^ method [83].

### 4.9. Statistical Analysis

Data are presented as mean and standard errors of the mean. Data were subjected to *t*-test and one-way ANOVA and Student–Neuman–Keuls multiple comparison tests in SPSS Statistics 22.0 (SPSS, Michigan Avenue, Chicago, IL, USA). *p* < 0.05 was considered statistically significant.

## 5. Conclusions

In summary, the results of the present study show that feeding 15% fat diets did not result in alterations in growth and feed utilization, but increased liver fat accumulation of groupers vs those subject to 10% fat diets. However, 1% taurine addition in a 15% fat diet not only improved its growth performance, but also reduced liver fat deposition. Liver transcriptome analysis showed that 49 DEGs were identified in the comparison of High–fat and Taurine groups, of which the expression of *CDO1*, *ATP1α*, *CAMK*, and *CACT* genes was significantly up-regulated and *ARF1_2* gene expression was significantly down–regulated. The key genes were involved in the taurine and hypotaurine metabolic pathway, bile secretion, insulin secretion, thermogenic pathway, and phospholipase D signaling pathway. As a result, the effect of dietary taurine on reducing liver fat accumulation of the fish species may be achieved by enhancing the synthesis of endogenous taurine in the liver, accelerating bile acid transport and promoting insulin secretion and fatty acid β-oxidation efficiency. The next step is to investigate the roles and functions of the key genes mentioned above in the fat metabolism of fish in response to dietary taurine.

## Figures and Tables

**Table 1:** Effects of experimental diets on growth performance and the contents of liver and muscle fat of groupers (*E. coioides*).

Parameters	Diets (Fat Level/Taurine Level)		
	Control (10/0)	High–Fat (15/0)	Taurine (15/1)
Weight gain (%)	151.44 ± 9.86 ^a^	157.38 ± 4.77 ^a^	180.07 ± 3.45 ^b^
Feed conversion ratio	0.99 ± 0.01 ^b^	1.00 ± 0.01 ^b^	0.91 ± 0.02 ^a^
Hepatosomatic index (%)	2.79 ± 0.14 ^b^	2.89 ± 0.06 ^b^	1.56 ± 0.24 ^a^
Liver lipid content (%)	9.57 ± 0.56 ^a^	13.38 ± 0.47 ^b^	9.05 ± 0.31 ^a^
Muscle lipid content (%)	1.31 ± 0.72 ^a^	1.95 ± 0.11 ^b^	1.79 ± 0.01 ^b^

Weight gain (%) = 100 × (final body weight − initial body weight)/initial body weight; Feed conversion ratio = feed intake/wet weight gain; Hepatosomatic index (%) = 100 × (liver weight/wet body weight); Data are presented as the means ± SEM (*n* = 3 tanks); Data was presented as the means ± SEM (*n* = 30 fish); Values in the same row with different lowercase letter superscripts indicate significant differences (*p* < 0.05); Statistical analysis was performed by one way ANOVA, followed by Student–Neuman–Keuls multiple comparison test.

**Table 2:** Summary statistics of the de novo transcriptome assembly of liver samples of *E. coioides*.

Items	Min Length	Max Length	Mean Length	N50	N90
Unigene number	200	16,130	532	653	243

Min length, the minimum sequence length in a unigene set; Max length, the maximum sequence length in a unigene set; Mean length, average sequence length of a unigene set; N50/N90, the unigenes were calculated by ordering all sequences, and the length of unigenes was then collected one by one from the longest to the shortest until 50%/90% of the total length was attained.

**Table 3:** The number of annotated genes in different databases.

Type of Database Annotation	Number of Unigenes	Percentage (%)
NR	72,125	20.65
SwissProt	13,104	3.75
PFAM	22,814	6.53
GO	6988	2.00
KO	5124	1.47
Annotated in all databases	487	0.14
Annotated in at least one database	72,679	20.81
Total Unigenes	349,211	100

NR, NCBI non-redundant protein sequence database; SwissProt, protein sequence database; PFAM, a large collection of protein multiple sequence alignments and profile hidden Markov models; GO, gene ontology database; KO, KEGG orthology database for representation of gene/protein functional orthologs in molecular networks; KEGG, kyoto encyclopedia of genes and genome database.

**Table 4:** Ingredients and proximate composition of experimental diets (on an as–fed basis, %) ^1^.

Ingredients	Diets (Fat Level/Taurine Level)		
	Control (10/0)	High-Fat (15/0)	Taurine (15/1)
Animal by–product (casein:gelatin = 4:1)	50	50	50
Shrimp meal	4	4	4
Corn starch	25	25	25
Oil (fish:soy oil = 1:1)	6	10	10
Soy lecithin	4	4	4
Premix	0.8	0.8	0.8
Ca(H_2_PO_4_)_2_	2	2	2
Microcrystalline cellulose	7.2	3.2	2.2
Sodium alginate	1	1	1
Taurine	0	0	1
Nutrient level (analyzed values)			
Dry matter	91.18	90.24	90.38
Crude protein	46.55	46.87	46.56
Crude lipid	10.44	14.79	14.89
Taurine	0.04	0.04	0.98

^1^ All feed ingredients are provided by Jiakang Feed Co., Ltd. (Xiamen, China).

## Data Availability

The data that support the findings of this study are available from the corresponding author upon reasonable request; further inquiries can be directed to NCBI Database at https://www.ncbi.nlm.nih.gov/bioproject/PRJNA853943 (accessed on 29 June 2022).

## Supplementary Materials

The following supporting information can be downloaded at: https://www.mdpi.com/article/10.3390/metabo12070670/s1. Figure S1: The sequence length of transcript and unigenes; Table S1: Alignment statistics of the RNA–Seq analysis of the nine liver samples of *E. coioides* between Control and High–fat groups, and between High–fat and Taurine groups; Table S2: The DEGs with significantly changed KEGG pathways related to lipid metabolism in the liver of *E. coioides* in the comparison of High–fat and Taurine groups; Table S3: Primers sequences of lipid metabolism related genes used for real–time PCR for *E. coioides*.
