# Supplementary material for: Hepatic Transcriptome Analysis Provides New Insight into the Lipid-Reducing Effect of Dietary Taurine in High–Fat Fed Groupers (Epinephelus coioides)
Source: Metabolites. 2022 Jul 20;12(7):670. doi: 10.3390/metabo12070670 (PMC9318954; doi:10.3390/metabo12070670)
Supplement: Supplementary file 1 [file metabolites-12-00670-s001.zip › metabolites-1827055-supplementary.pdf]

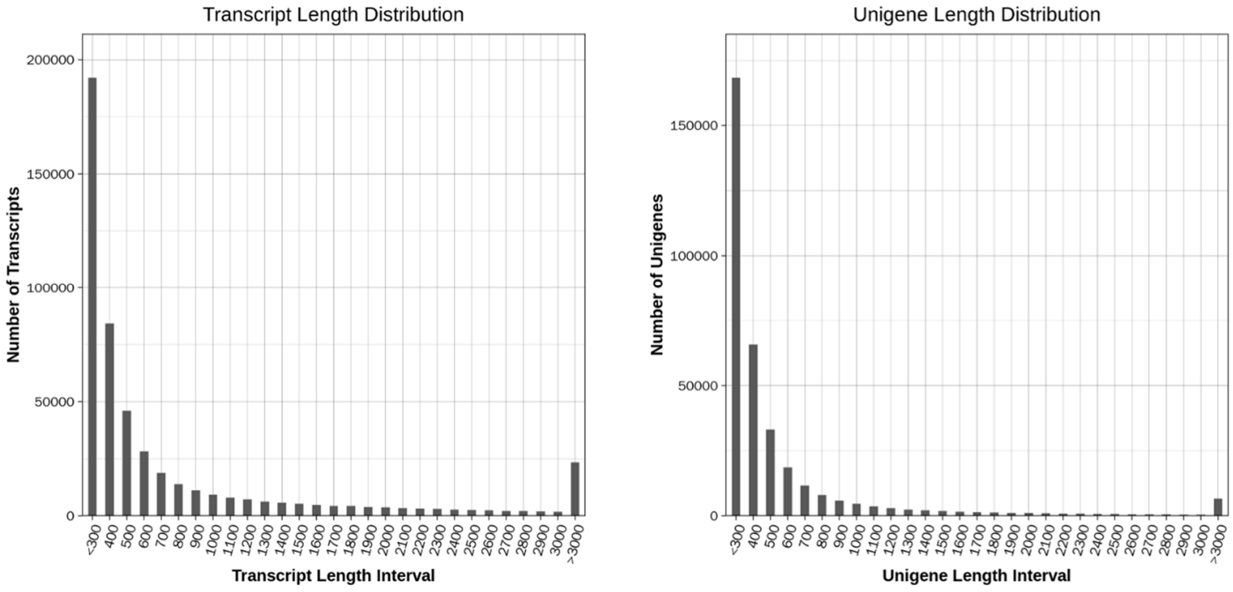

Figure S1. The sequence length of transcript and unigenes

The sequence length statistics of transcript and unigenes in the liver samples of *E. coioides*.

The horizontal axis represents the sequence length interval, and the vertical axis represents the number of transcripts and unigenes corresponding to the sequence length interval.

Table S1. Alignment statistics of the RNA−Seq analysis of the nine liver samples of *E. coioides* in Control, High−fat and Taurine groups

Sample	Raw reads	Clean reads	Error (%)	Q20 (%)	Q30 (%)	GC (%)
Control-1	40730652	40050010	0.03	97.79	93.83	43.39
Control-2	42191002	41722212	0.03	97.82	93.79	43.99
Control-3	40948846	40336514	0.03	97.8	93.82	42.16
High-fat-1	52582890	52119034	0.02	98.07	94.3	45.84
High-fat-2	52479728	52027176	0.02	98.46	95.45	43.35
High-fat-3	40644396	40196728	0.02	98.14	94.53	47.00
Taurine-1	46268020	45571324	0.03	97.56	93.16	45.94
Taurine-2	46244384	45663502	0.03	97.83	93.69	46.84
Taurine-3	41988098	41563038	0.03	97.94	94.09	45.03

Control 1−3, triplicate groups fed with control diet; High−fat 1−3, triplicate groups fed with 15% fat diet; Taurine 1−3, triplicate groups fed with 15% fat and 1% taurine. Raw reads, unfiltered Reads were off the machine; Clean reads, the total number of Clean reads.

Table S2. The DEGs with significantly changed KEGG pathways related to lipid metabolism in the liver of *E. coioides* in the comparison of High−fat and Taurine groups

Gene ID	Gene description	Gene name abbreviations	Pathway	Log_2FC	RNA−seq Expression Pattern	qRT−PCR Expression Pattern
TR4334_c0_g1	ADP−ribosylation factor 1/2	ARF1_2	Phospholipase D signaling pathway	−6.527	down	down
TR255_c0_g1	pyruvate dehydrogenase kinase isozyme 2	*PDK1*	HIF−1 signaling pathway	−2.739	down	up
TR41649_c0_g1	glucokinase	*GK*	Glycolysis /Gluconeogenesis	−7.224	down	down
TR669_c0_g1	cysteine dioxygenase	*CDO1*	Taurine and hypotaurine metabolism	2.184	up	up
TR7788_c3_g1	sodium/potassium-transporting ATPase subunit alpha	*ATP1α*	Bile secretion/ Insulin secretion	2.379	up	up
TR62_c0_g1	calmodulin−dependent protein kinase II	*CAMK*	Insulin secretion	9.198	up	up
TR10507_c0_g1	phosphatidylinositol phospholipase C	*PLCD*	Inositol phosphate metabolism / Phosphatidylinositol signaling system / Calcium signaling pathway	1.978	up	down
TR4858_c0_g1	carnitine/acylcarnitine translocase	*CACT*	Thermogenesis	3.156	up	up

log2 FC, unigenes with |log2 (Fold Change)| > 1.0.

Table S3. Primers sequences of lipid metabolism related genes used for real−time PCR for *E. coioides*

Target Gene	Forward (5′−3′)	Reverse (5′−3′)	Accession number
β−action	TGCTGTCCCTGTATGCCTCT	CCTTGATGTCACGCACGAT	AY510710.2
ARF1_2	GTTCAGGTCTGGTCGAGGGT	TCAGGTCTCAGCAAACTGCC	NM_201452.2
PDK1	AGGGCAACACAAAAAGCGGA	GCGTGCCAACTGCCATCTAT	NM_200996.1
GK	GCCCTCTGTTCATCTCACGC	TGAAGATGCCGTGTGTCAGC	NM_001045385.2
CDO1	GTGCCAAACAGCATCGGAGA	AGGTTTTCACCAGGAGCCGA	NM_200741.1
ATPase	CCAGTTACCACGACGACAGC	GTGTCTGATGTGGGTCGCAG	NM_131686.1
CAMK	TGGAGTGTGTGTTTTGGGGG	TATCGTGGAGAGGGATGCGA	GH643012.1
PLCD	ATCCCGTTTCTGGGGTGTCT	AATGTGGAATGCTGGGTGCC	NM_001122773.1
CACT	GCAATGAGACCTTGGAGGCT	TTGAAGTCGGTGAGAAGCCC	BC062851.1

Abbreviations: *ARF1_2*, ADP−ribosylation factor 1/2; *PDK1*, pyruvate dehydrogenase kinase isozyme 2; *GK,* glucokinase; *CDO1*, cysteine dioxygenase; *ATP1α*, sodium/potassium−transporting ATPase subunit alpha; *PLCD*, phosphatidylinositol phospholipase C; *CACT*, the carnitine/acylcarnitine translocase; *CAMK*, calcium/calmodulin−dependent protein kinase (CaM kinase) II.
